# Evaluation of malaria prevention strategies during pregnancy in Ndola, Zambia

**DOI:** 10.4102/phcfm.v2i1.159

**Published:** 2010-11-09

**Authors:** Mwamba Mulamba, Bob Mash

**Affiliations:** 1 Division of Family Medicine and Primary Care, Stellenbosch University, South Africa

**Keywords:** malaria, pregnancy, prevention, sulfadoxine/pyrimethamine, Zambia

## Abstract

**Background:**

Malaria in pregnancy is associated with many negative outcomes for the woman, foetus and neonate. Intermittent preventive treatment during pregnancy (IPTp) using three doses of sulfadoxine/pyrimethamine (SP), insecticide-treated mosquito nets (ITNs) and indoor residual spray (IRS), constitute the main strategies used to prevent malaria. The aim of this study is to evaluate the effectiveness of these strategies for the reduction of malaria prevalence in pregnant women.

**Methods:**

A questionnaire on socio-demographic information, history of malaria during current pregnancy and prevention strategies used was administered to 450 consecutive patients admitted into labour wards at three local clinics. From the antenatal cards, information was collected on the last menstrual period, date of each dose of SP taken, gravidity, and HIV status. A blood slide to detect *Plasmodium* was then collected from each woman after consent.

**Results:**

Of the participants in the study, 2.4% had a positive blood slide at term and 15.8% reported malaria during pregnancy. All the participants took at least one dose of SP with 87.6% completing the stipulated three doses. The mean gestational ages for each dose were 22.1 (SD 4.6), 29.1 (SD 4.4) and 34.4 (SD 3.9) weeks for the first, second and third dose respectively. With regard to ITNs, 79.5% had one, but only 74.1% used it regularly. IRS was completed in all three of the clinics’ catchment areas. Only 23.4% used commercial insecticide.

**Conclusion:**

The measured prevalence of malaria at term in Ndola was remarkably low, although the self-reported rate during pregnancy was still high. The national targets for accessing IPTp were exceeded, although the timing of each dose needs to be improved. Access to ITNs was high, but usage needs to increase.

## INTRODUCTION

Malaria infection due to *Plasmodium falciparum* still causes significant morbidity and mortality in countries with limited resources that are situated in endemic zones. It is still a global health problem, with most of the disease burden carried by children younger than 5 years of age and pregnant women.^1^ It is estimated that up to 90% of global cases of malaria occur in sub-Saharan Africa.^1, 2^

Although there are four species causing malaria, most complications are due to the *P. falciparum* species.^1–3^ In pregnancy, malaria poses a risk to the mother, the foetus and the neonate. In areas of stable malaria where adult women have considerable acquired immunity, *P. falciparum* infection during pregnancy does not typically develop into a symptomatic febrile disease, but rather leads to maternal anaemia and placental malaria infection, particularly in the primigravidae and the secundigravidae.^1–4^ On the other hand, pregnant women who are HIV-positive are particularly vulnerable to malaria.^5–8^ *P. falciparum* is associated with an increased risk of maternal anaemia and mortality. It is also accompanied by an increased risk of prematurity.

Placental malaria can lead to cord anaemia, intrauterine growth retardation, low birth weight (LBW), and possibly intrauterine death. Neonatal malaria is also possible and leads to an increased risk of infant mortality.^1, 9, 10^

All of these complications can be avoided by cost-effective prevention of malaria in pregnancy, as well as early detection and effective treatment of patients.

Zambia is a southern African country where malaria is endemic and still constitutes a major public health problem. The Zambian government has designated malaria control as one of its priorities, as demonstrated in the National Health Strategic Plan 2006–2010.^11^ It is estimated that in Zambia, 3.4 million cases of malaria occur every year, translating into 308 infections per 1000 persons each year and that sulfadoxine/pyrimethamine (SP) total treatment failure has risen from 14.5% in 2003 to 37.0% in 2004.5 The HIV prevalence in Zambia is 16.0% amongst adults aged 15–49 years, while amongst pregnant women attending antenatal care, the prevalence is 19.0%.^12^

Currently, efforts to control malaria in Zambia are being escalated and coordinated under the Roll Back Malaria (RBM) initiative. Under this initiative, the Zambian government, in collaboration with other partners, has come up with four strategies, namely (1) use of insecticide-treated mosquito nets (ITNs), (2) indoor residual spray (IRS) in all houses, with pyrethroids and dichlorodiphenyltrichloroethane (DDT), (3) use of intermittent preventive treatment of malaria in pregnancy (IPTp) and (4) prompt, effective case management.

IPTp was introduced in 2003 and requires three doses of an SP combination that are given to pregnant women at the gestational ages of 16, 20 and 24 weeks during their antenatal care visits.^13^ Though studies elsewhere advise monthly SP for HIV-positive pregnant women,^14, 15^ a 2007 Zambian study concluded that in zones of meso-endemic malaria transmission, this was not more efficacious than the standard regimen.^5^

The use of SP in pregnancy as IPTp has been demonstrated to be safe, however, its concomitant use with co-trimoxazole or with high-dose folic acid is not advisable.^16^ The implication of this is crucial for HIV-positive women who are on co-trimoxazole prophylaxis.

At the antenatal visits, ITNs are also distributed at a very subsidised price of $1.00 per net and health talks about the effects of malaria on pregnancy are communicated. IRS spraying at home is provided through the District Health Management Team (DHMT).

The Ministry of Health recommends that pregnant women diagnosed with malaria be treated with quinine only in the first trimester, or if the malaria is severe. In the second and third trimesters, SP should be prescribed. The treatment should commence within 24 hours of the onset of symptoms.^11, 13^

Although the Zambian malaria control programme in pregnant women was implemented in 2003, its success has not been widely studied. The aim of this study was to evaluate the effectiveness of strategies employed to prevent malaria in pregnant women in Ndola, Zambia and, if possible, to make recommendations on how prevention can be improved.

The specific objectives were:

- To determine the proportion of pregnant women who have access to and complete the recommended three-dose course of IPTp.
- To determine the gestational age at which each dose of IPTp is taken.
- To determine the prevalence of maternal parasitemia at term in pregnant women taking SP/IPTp.
- To determine the proportion of pregnant women who sleep under ITNs regularly.
- To describe the provision of IRS and other non-official strategies used by pregnant women to prevent malaria.
- To determine what type of medication is prescribed to pregnant women when they contract malaria.

## ETHICAL CONSIDERATIONS

The study was approved by the Human Research Ethics Committee, Stellenbosch University, South Africa and the Ethics Committee, Tropical Disease Research Centre, Zambia.

## METHODS

A cross-sectional study evaluated the effect of the RBM prevention strategy on the prevalence of malaria in pregnancy within the maternity ward.

### Settings

The study was conducted in three health clinics in the suburb of Ndola between January and April 2009. Ndola is one of the urban centres in the Copperbelt province of Zambia, in southern Africa. These three clinics provide health care to approximately 96 000 people. In all three clinics women give birth routinely and the strategies for malaria prevention in pregnancy follow the national programme. This includes sleeping under an ITNs, receiving three doses of SP and yearly IRS with pyrethroids and dichlorodiphenyltrichloroethane (DDT), which is carried out by the Ndola DHMT.

Every woman attending antenatal classes is educated on HIV and prevention of mother-to-child transmission. Zambia uses the opt-out strategy when it comes to HIV testing in pregnant women. The HIV status is then coded on the antenatal card and on the under-5 card of the child after birth.

### Sample size

Taking into consideration that in the chosen three clinics, the total average number of deliveries is 7000 per annum and the expected malaria prevalence at delivery is 13.6% without IPTp and 6.3% after IPTp.^17^ For the purpose of the study a minimum sample size of 350 was calculated using Cochrane's cross-section survey sample size calculation.

### Enrolment

Participants who had given informed consent and were at least 28 weeks pregnant and 18 years or older, were enrolled consecutively as they came into the maternity ward. We excluded those with (1) a history of splenectomy, (2) sickle cell disease, (3) allergy to sulfadoxine/pyrimethamine and (4) those that came to the maternity ward without the antenatal cards. Enrolment into the study ceased once 450 patients had consented.

### Questionnaire procedures

The purpose of the study and its requirements were explained to the patients prior to obtaining written, informed consent from each participant. Enrolled women were given a questionnaire by the local midwife to collect (1) socio-demographic information, (2) history of malaria during the current pregnancy, (3) any anti-malarial treatment and (4) malaria prevention strategies used. From each participant's antenatal card, information was collected (1) on the last menstrual period, (2) date at which each dose of IPTp was taken, (3) gravidity and (4) HIV status.

### Laboratory procedures

A drop of blood was collected using a fingerprick sample, and a blood slide was prepared and air-dried. The slides were stained with Giemsa stain and then examined at the Tropical Disease Research Centre twice a week by a laboratory technician. The number of parasites per 200 white blood cells determined the parasite count and a slide was only considered negative after 100 oil-immersion fields were examined and no parasite found to be present.

## RESULTS

Out of the 450 participants (150 from each clinic), 11 (2.4%) blood slides tested positive for malaria parasite and 439 (97.6%) tested negative. Their characteristics are shown in Table 1.

**TABLE 1:** Characteristics of enrolled women participants

Characteristics	*n*	%
**Marital status**		
single	20	4.4
married	430	95.6
**Total**	**450**	-
**Gravidity**		
primigravidae and secundigravidae	122	27.4
multigravidae (three or more)	323	72.6
**Total**	**445**	-
**Education level**		
primary school (Grades 1–7)	153	34.5
secondary school (Grades 8–12)	281	63.4
tertiary school (college or university)	9	2.0
**Total**	**443**	-
**HIV status**		
positive	89	20.0
negative	356	80.0
**Total**	**445**	-
**IPT**†		
no	0	0.0
yes (any dose)	450	100.0
one dose	4	0.9
two doses	52	11.6
three doses	394	87.6
**Total**	**450**	-
**ITN use**†		
no	92	20.5
yes	356	79.5
regularly (four or more times per week)	332	93.3
occasionally (twice or three times per week)	16	4.5
seldom (once or less per week)	8	2.2
**Total**	**448**	-
**Commercial insecticide use**		
no	343	76.6
yes	105	23.4
regularly (four or more times per week)	35	33.3
occasionally (twice or three times per week)	33	31.4
seldom (once or less per week)	37	35.2
**Total**	**448**	-
**Repellents use**		
no	443	98.9
yes	5	1.1
regularly (four or more times per week)	0	0.0
occasionally (twice or three times per week)	3	60.0
seldom (once or less per week)	2	40.0
**Total**	**448**	-
**Blood slide results**		
positive	11	2.4
negative	439	97.6
**Total**	**450**	-
		

† IPT = intermittent preventive treatment; ITN = insecticide-treated mosquito net.

*n* = given as means of value.

Information about the gravidity of five participants was absent. Of the 445 for whom gravidity information was available, 122 (27.4%) were either primigravidae or secundigravidae. This represents the group of pregnant women who are at a higher risk for adverse outcomes.

Out of the 445 participants who revealed their HIV status, 89 (20.0%) were HIV-positive. Of these, 15 (16.9%) were either primigravidae or secundigravidae. This means that 15 women out of the 445 were both HIV-positive and either primigravidae or secundigravidae, representing 3.4%.

Of the 450 participants, all had taken at least one dose of SP (100.0%), and 394 (87.6%) had completed the course of all three doses. Overall, 99.1% of participants took two or three doses of IPTp.

The mean gestational ages for each dose were 22.1 (SD 4.6), 29.1 (SD 4.4) and 34.4 (SD 3.9) weeks for the first, second and third doses respectively. Only 18 participants (4.1%) took the first dose at 16 weeks, 3 (0.7%) took the second dose at 20 weeks and 1 (0.3%) took the third dose at 24 weeks, as recommended in the national guidelines. In addition, 14.9% of the women who took three doses of IPTp received their last dose after 38 weeks of gestation.

We found that 448 participants owned ITNs; of these, only 356 (79.5%) actually used them. The results also show that 332 women (73.8%) used their ITNs regularly (four times a week or more).

Information collected about the use of commercial insecticide showed that 105 respondents (23.4%) had used it. Only 35 (7.8%) used it regularly; this means that the majority of our participants (92.3%) either used it occasionally, or not at all. More importantly, 76 (72.4%) women out of the 105 still used ITNs regularly. Only five participants (1.1%) applied any mosquito repellents.

Although only 2.4% of women were positive for malaria when tested (Table 1), 15.8% reported that they had malaria during their pregnancy and received treatment, as shown in Table 2.

**TABLE 2:** Self-reported malaria and treatment received

Variable	*n*	%
**Malaria**		
no	377	84.2
yes	71	15.8
**Total**	**448**	-
**Curative medication received**		
artemisinin	1	1.4
SP†	44	62
quinine	26	36.6
**Total**	**71**	-

† SP, sulfadoxine/pyrimethamine.

Almost two-thirds of women who suffered from malaria were treated with SP, while only a third (36.6%) received quinine.

Out of the 11 women who had a positive slide, 5 were HIV-positive, representing 45.5% of the positive slides. Of the 89 HIV-positive participants, these 5 suggest a prevalence of 5.6%, which is more than double the baseline value.

The DHMT confirmed that IRS spraying had been performed throughout the catchment areas of all three clinics during the previous year.

## DISCUSSION

### Main findings of this study

Overall, the RBM programme was implemented effectively, with 87.6% of pregnant women receiving three doses of SP, 79.5% owning an ITNs and households in all areas being offered IRS. The timing of the three doses of SP was not aligned with the guidelines and only 74.1% used their ITNs regularly. Only a small number of women supplemented the official strategies with the use of their own commercial insecticides and repellents. The prevalence of malaria in the maternity ward was low at 2.4%, whilst the self-reported prevalence during pregnancy was 15.8%.

### Comparison to previous studies

The prevalence of malaria in this study is significantly lower than the 6.3% found in Mozambique (with two doses of SP as the prevention strategy).^17^ The difference can be explained by the fact that in the Zambian situation, many other strategies are simultaneously implemented, such as IRS and ITNs, as part of the Integrated Vector Control Programme, in conjunction with the prompt treatment of infected individuals.

Although it was found that 15.8% of participants reported having suffered from malaria during their current pregnancy, many people suffering from any febrile illness in an endemic region like Zambia would call it malaria and a large number will self-medicate.^18^ In addition, most pregnant women who present at their local clinic with fever are likely to be told that they are suffering from malaria and receive treatment with anti-malarial medication. Many health care workers still prescribe anti-malarial treatment, even when the malaria test is negative.^18^ Nonetheless, this percentage is similar to the 15.7% malaria parasitemia found in Burkina Faso.^4^ In this case, the study was done in a more rural area, the prevention strategy did not include regular IRS, and the IPTp progamme had only been running for 1 year. On the other hand, the fact that malaria in pregnancy is usually asymptomatic means that many pregnant women with no fever do not seek medical advice, even if they may have malaria. The true prevalence of malaria in pregnancy, therefore, can only be fully assessed through placental biopsy. Although the levels of total treatment resistance to SP have increased over the past few years, its effectiveness as an intermittent preventive treatment is still high.^14^ It is also possible that the use of IPTp as an integrated part of a larger strategy including other measures, such as ITNs, increases the effectiveness.

The Zambian Ministry of Health (MoH) and the World Health Organization (WHO) recommend quinine as the drug of choice during the first trimester and in severe malaria in pregnancy, while SP can be prescribed in the second and third trimesters.^1, 13^ However, with the current SP resistance level in Zambia,^19^ cost-effectiveness studies should be conducted with a view to prescribing quinine throughout pregnancy. The WHO actually recommends that in areas of combined resistance to chloroquine and SP, quinine should be the first-line drug, even in second and third trimester.^1^ In addition, studies should be done, locally, to assess the possible use of artemisinin and artemisinin combination therapies (ACT). Currently, Zambia does not recommend prescription of ACT during pregnancy, as there is no evidence base to show its safety. There is emerging data showing that artesunate and ACT are safe and efficacious in pregnancy.^20^ Further studies are required to evaluate alternatives to SP in the light of increasing resistance.

The use of IPTp met the goal of the Zambian MoH to ensure access to IPTp for at least 80% of women by December 2008. The level exceeds the findings of the Zambia National Malaria Survey in 2006, which recorded that 85.9% of urban women accessed any IPTp and only 71.2% took all three recommended doses.

On average, the first dose was received 6 weeks later than recommended; the second dose was 7 weeks after the first, and the third dose 5 weeks after the second; instead of the recommended 4-week interval. This late delivery certainly increases the time that pregnant women spend at risk. In addition, there are suggestions that the late delivery of the first dose of IPTp could be related to the subsequent late delivery of the other following doses, or lead to an incomplete IPTp schedule.^21^ Infection in early or late pregnancy has been associated with more negative effects on the pregnancy outcome^22^ and this fact should be the basis for advocating that the first dose of IPTp be on time.

The use of ITNs is not far below the target of 80% of pregnant women accessing ITNs by December 2008, as stated in the National Health Strategic Plan 2006–2010.^11^ Though the document is silent about the target concerning the use of these nets, we would want to believe that the intention of the document was to achieve a comparable target. This means that the current usage rate (74.1%) is well below the expected rate. Nonetheless, there has been tremendous improvement when compared to 18% of urban women who slept under an ITNs in 2006.^23^

Application of insect-repellent ointments or lotion is not a practice that is commonly embraced by the population. However, there is evidence to show that insect repellent in addition to ITNs provides greater protection, especially in areas where mosquitoes feed in the early evening, and between dusk and bedtime.^24^ In countries with restricted economic power, this might not be a cost-effective programme, but one which is particularly advocated for tourists and those who can afford it. However, more studies are needed to clarify their safety profile in pregnancy.

### Limitations

The exact prevalence of malaria during the whole of pregnancy could not be determined by this cross-sectional study within the maternity ward. These women were also likely to have frequented the same clinics for their antenatal visits and would most likely have been educated about the risk factors related to pregnancy in general and malaria in particular. Therefore they were more likely to have received IPTp and to use ITNs. The results, therefore, for the whole community of pregnant women, which includes those giving birth at home, those not attending the clinic and those in rural areas, are likely to be worse. The number of women refusing to participate in the study was not recorded by the research assistants and the uptake of IRS per household was not confirmed in the questionnaire. The questionnaire could have attempted to verify the gestational age of reported malaria and use of diagnostic tests, although this would still have been subject to a potential reporting and recall bias.

### Recommendations

Further studies should attempt to determine the rates of malaria throughout pregnancy and to assess if the self-reported rate of 15.8% is correct.

There is a need to improve the gestational age at which the first dose of IPT is delivered, as well as the follow-up doses. This can be achieved through education of health care providers and community sensitisation by health talks given during antenatal classes.

Although access to ITNs has improved dramatically, attention must now be given to the usage rate. There is a need for more educational messages to pregnant women about the dangers of malaria, the emphasis on taking IPT on time, and always sleeping under ITNs, especially if one is HIV-positive. The correct treatment of malaria in pregnancy as well as the risks of over-prescription of anti-malaria drugs needs to be emphasised to health care workers, during continuing medical education sessions. More research needs to be conducted to evaluate the situation in rural areas and amongst those women who give birth at home with the help of traditional birth attendants.

## CONCLUSION

The aim of this study was to evaluate the effectiveness of strategies employed to prevent malaria in pregnant women in Ndola, Zambia, and if possible to make recommendations on how prevention can be improved. The study evaluated 450 consecutive pregnant women admitted into maternity wards at three local clinics. Overall, the Rolling Back Malaria programme was implemented effectively, with 87.6% of pregnant women receiving three doses of SP, 79.5% owning an ITNs and households in all areas being offered IRS. The timing of the three doses of SP was not aligned with the guidelines and only 74.1% used their ITNs regularly. Only a small number of women supplemented the official strategies with the use of their own commercial insecticides and repellents. The prevalence of malaria in the maternity ward was low at 2.4%, whilst the self-reported prevalence during pregnancy was 15.8%. The results show that the programme is on target for use of IPTp, although improvements can be made on the timing of SP doses and the actual usage of the ITNs.
